# Diabetic foot

**DOI:** 10.1136/bmj.j5064

**Published:** 2017-11-17

**Authors:** Satish Chandra Mishra, Kunal C Chhatbar, Aditi Kashikar, Abha Mehndiratta

**Affiliations:** 1 Department of Surgery, Bhabha Atomic Research Centre Hospital, Mumbai, India; 2 KHM Hospital, Mumbai, India; 3 Seth Gordhandas Sunderdas Medical College and King Edward Memorial Hospital, Mumbai, India; 4 Global Health and Development Group, Imperial College London, St Mary’s Hospital, London, UK

**What you need to know**

- Diabetic foot can be prevented with good glycaemic control, regular foot assessment, appropriate footwear, patient education, and early referral for pre-ulcerative lesions
- Examine the feet of people with diabetes for any lesions and screen for peripheral neuropathy and peripheral arterial disease, which can lead to injuries or ulceration
- Refer patients with foot ulceration and signs of infection, sepsis, or ischaemia immediately to a specialised diabetic foot centre for surgical care, revascularisation, and rehabilitation

Foot disease affects nearly 6% of people with diabetes^1^ and includes infection, ulceration, or destruction of tissues of the foot.^2^ It can impair patients’ quality of life and affect social participation and livelihood.^3^ Between 0.03% and 1.5% of patients with diabetic foot require an amputation.^4^ Most amputations start with ulcers and can be prevented with good foot care and screening to assess the risk for foot complications.^5^ We provide an update on the prevention and initial management of diabetic foot in primary care.

**Sources and selection criteria**

This clinical update is based on recommendations in the standard treatment guideline, The diabetic foot: prevention and management in India 2016, published by the Indian Ministry of Health and Family Welfare.^33^ A multidisciplinary guideline development group consisting of surgeons, primary care practitioners, and a patient representative developed these guidelines, with inputs from experts in diabetes, diabetic foot rehabilitation, and vascular surgery. The group included representation from rural and urban India, and public and private sectors.

The guideline development group selected recommendations from the National Institute for Health and Care Excellence clinical guideline 19. Diabetic foot problems: prevention and management. Updated 2016, International Working Group on the Diabetic Foot guidance on the prevention of foot ulcers in at-risk patients with diabetes 2015, National Institute for Health and Care Excellence. Peripheral arterial disease: diagnosis and management. Guideline 147, 2012, and Infectious Diseases Society of America clinical practice guideline for the diagnosis and treatment of diabetic foot infections, 2012.^9^ ^10^ ^21^ ^32^ Some recommendations were adopted unchanged, whereas others were adapted taking into account the challenges of a low resource setting, such as availability of public and private health infrastructure, equipment, staffing, and current capacity at different levels of care.

## What causes diabetic foot?

Uncontrolled diabetes contributes to the development of neuropathy and peripheral arterial disease by complex metabolic pathways.^6^ Loss of sensation caused by peripheral neuropathy, ischaemia due to peripheral arterial disease, or a combination of these may lead to foot ulcers. A systematic review (78 studies from 84 cohorts) reports a prevalence of 0.003-2.8% for diabetes related peripheral neuropathy and 0.01-0.4% for diabetes related peripheral arterial disease.^4^ Figure 1 [Fig f1]depicts factors that contribute to foot complications.

**Figure f1:**
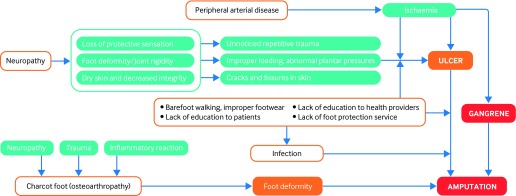
**Fig 1** Risk factors and mechanism for foot ulcer and amputation

Diabetes is also implicated in Charcot arthropathy, which involves progressive destruction of the bones, joints, and soft tissues, most commonly in the ankle and foot. Diabetes related Charcot’s arthropathy has a reported prevalence between 0.08% and 13%, but there are no high quality epidemiological studies on Charcot’s foot.^7^ ^8^ A combination of neuropathy, abnormal loading of foot, repeated micro trauma, and metabolic abnormalities of bone leads to inflammation, causing osteolysis, fractures, dislocation, and deformities.

In low and middle income countries barefoot walking, lack of awareness, delay in seeking care, and shortage of trained healthcare providers and foot care services are common factors that add to the burden of foot disease.

## How is it diagnosed?

A thorough foot examination is important to detect the disease early. Screening for peripheral neuropathy and peripheral arterial disease can help identify patients at risk of foot ulcers. A history of ulcers or amputations and poor glycaemic control increase the risk.

Assess the patient’s general condition for signs of toxicity or sepsis such as feeling unwell, looking sick, showing abnormal behaviour, circulation, or respiration, with or without fever. Examine the feet at each follow-up visit for active disease such as ulceration or gangrene (fig 2). Look for lesions such as fungal infection, cracks and skin fissures, deformed nails, macerated web spaces, calluses, and deformities such as hammer toes, claw toes, and pes cavus, which increase the risk of ulceration (fig 3). Feel the temperature of the feet with the dorsum of your hand. A cold foot might suggest ischaemia, and increased warmth with redness and swelling might suggest inflammation such as acute Charcot foot or cellulitis.

**Figure f2:**
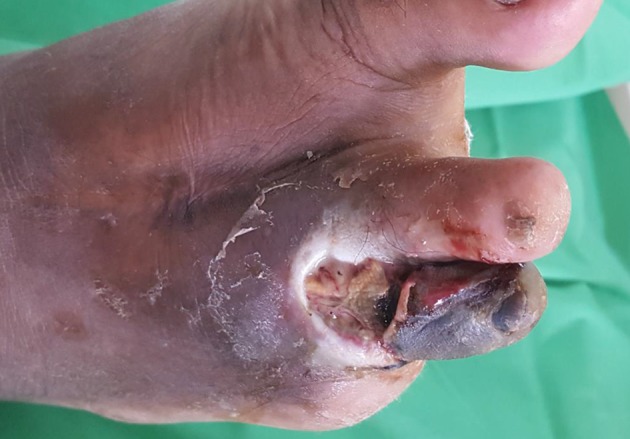
**Fig 2** Gangrene and ulcer in foot at high risk (previous toe amputation)

**Figure f3:**
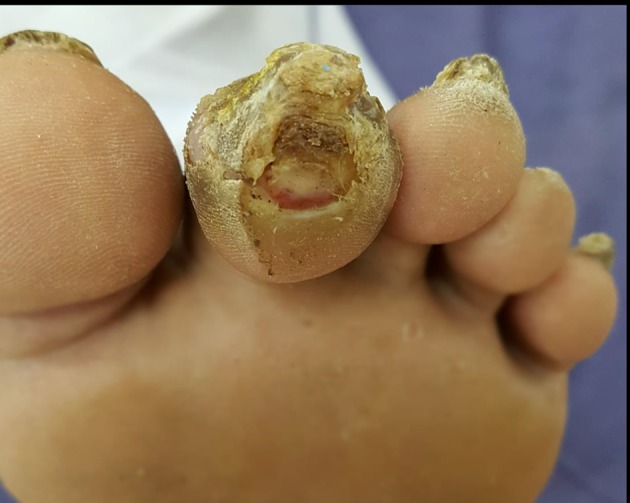
**Fig 3** Hammer toe deformity with callus and ulcer. Hammer toe is caused by weakened muscles in the foot. The joint connecting the foot with the toe bends upwards (metatarsophalangeal extension) and the joint in middle of the toe bends downwards towards the floor (proximal interphalangeal flexion). This results in the toe curling under the foot and being subjected to excessive ground reaction forces during walking.

### Peripheral neuropathy

The aim of screening is to identify patients with loss of protective sensation in the feet. Most guidelines recommend the 10 g monofilament for neuropathy assessment (fig 4) in people with diabetes.^9^ ^10^ This monofilament exerts a 10 g buckling force when it bends. An inability to sense a 10 g pressure is the current consensus definition of loss of protective sensation. The test is portable, cheap, and easy to perform (box 1).^12^ ^15^ Despite the widespread use of the monofilament test, its accuracy in diagnosing neuropathy is variable.^16^ The test may be combined with another test to screen for neuropathy, such as a biothesiometer or a graduated tuning fork (Rydel Seiffer) to assess vibration perception threshold.^17^ ^18^

**Figure f4:**
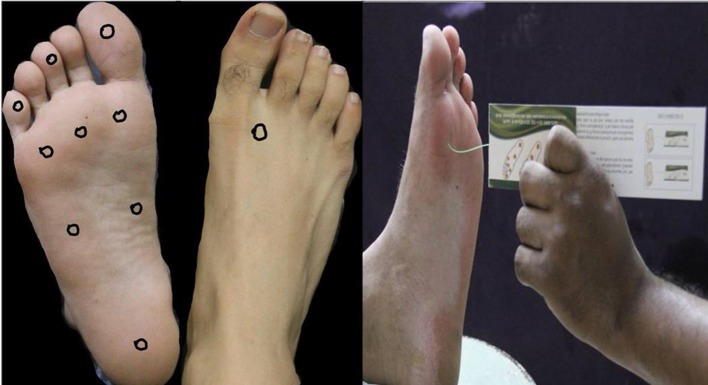
**Fig 4** Monofilament test: testing sites and application. The nine plantar sites are the distal great toe; third toe; fifth toe; first, third, and fifth metatarsal heads; medial foot, lateral foot, and heel; and one dorsal site

**Box 1: Monofilament test (fig 4)**

*Procedure*—Ask the patient to sit or lie down with both legs stretched out and soles exposed. Explain the procedure and make him or her familiar with the sensation by applying the monofilament on a sensitive area such as the palm. Ask the patient to close his or her eyes and to say “yes” every time touch is felt on the soles, no matter how lightly it is perceived. Place the monofilament at 90° to the skin and press it till it buckles to 1 cm, then hold there for 1-2 seconds and remove.^11^ Test different sites in a random sequence with a pause (sham application) to prevent the patient from guessing the next application. If the patient fails to respond at a site, revisit the same site two more times in a random sequence during the assessment. If the patient does not perceive the sensation all the three times, then record the result as loss of protective sensation.^11^ Loss of protective sensation even at a single site puts the patient at risk for foot complications.

*Test sites and threshold*—Most studies recommend testing at 10 sites.

Inability to perceive a 10 g monofilament three times at even a single site means the patient has loss of protective sensation.^11^ ^12^

*Inter-observer variability*—This is reported to be more on the heels, with a higher chance of a false positive result.^13^ Exercise caution before labelling a heel as insensate, especially if screening a population where barefoot walking is common.

*Durability of monofilaments*—Monofilaments tend to fatigue with repeated use, and a 24 hour recovery period is recommended after 100 compression cycles.^14^ Replace a monofilament after three months of regular use.

### Peripheral arterial disease

Ask for a history of intermittent claudication and rest pain, which suggest peripheral arterial disease.^19^ Palpate the posterior tibial artery and dorsalis pedis artery in both feet and record pulsations as absent or present.^20^

The ankle brachial index is an adjunct measure to diagnose peripheral arterial disease.^19^ ^21^ It is the ratio of the highest systolic blood pressure at the ankle (dorsalis pedis artery or posterior tibial artery) to the systolic blood pressure at the arm, and is measured using a Doppler device.^10^ See box 2 on grading the severity of obstruction. Measurement of the ankle brachial index is user dependent. People with diabetes can often have falsely raised ankle brachial index levels as a result of poor compressibility from calcified arteries.^21^ Furthermore, availability of equipment, time constraints, and lack of training are reported as major barriers to ankle brachial index testing in primary care.^23^ ^24^ ^25^

**Box 2: Ankle brachial index**

The severity of peripheral arterial disease is interpreted^22^:

- 0.91-1.3—Normal
- 0.70-0.90—Mild obstruction
- 0.40-0.69—Moderate obstruction
- <0.40—Severe obstruction
- >1.3—Poorly compressible vessel

On the basis of this initial assessment, patients can be categorised as having a low, moderate, or high risk of diabetic foot (see infographic).^9^

## How can it be prevented?

### Regular foot examination

The suggested frequency for follow-up is based on expert consensus (see infographic). For people at low risk, continue annual foot assessments as they could progress to moderate or high risk. Emphasise the importance of foot care and monitoring glycaemic control.

More frequent follow-up is advised in patients at moderate or high risk, such as those with a foot deformity or with a diagnosis of peripheral neuropathy or peripheral arterial disease at initial assessment. Repeat testing for neuropathy is not necessary if diagnosed previously. Neuropathy reversal is not established in studies. A quick inspection for a breach in skin integrity or ulceration should suffice. Patients with asymptomatic peripheral arterial disease may be followed up in primary care and managed as in guidelines for peripheral arterial disease.^21^

Refer patients with calluses and deformed toe nails to preventive podiatry services for basic nail and skin care, including debridement of calluses. Timely referral to foot protection services for control of risk factors in patients with diabetes prevents infection, gangrene, amputation, or death, and reduces hospital admissions and costs.^9^

### Glycaemic control

Early and good glycaemic control is effective in preventing neuropathy but there is a lack of studies to show that glycaemic control reverses neuropathy.^26^ Discuss optimal blood sugar and glycated haemoglobin (HbA_1c_) targets with patients and monitor these as per standard guidelines for diabetes care to prevent or slow the progression of peripheral neuropathy.^27^ ^28^

### Patient education

Offer people with diabetes or their caregivers, or both, oral and written information on:

- The importance of blood glucose control and modifiable cardiovascular risk factors such as diet, exercise, body weight, and cessation of smoking.
- The importance of foot care and advice on basic foot care (see box 3). While offering advice consider the patient’s cultural practices and religious beliefs as well as social and family support.
- The person’s current risk of developing a foot problem.
- When to seek professional help and who to contact in foot emergencies.

**Box 3: Tips on foot care for people with diabetes^19^**

- Inspect both feet daily, including the area between the toes. Ask a caregiver to do this if you are unable to.
- Wash the feet daily with water at room temperature, with careful drying, especially between the toes.
- Use lubricating oils or creams for dry skin, but not between the toes.
- Cut nails straight across.
- Do not remove corns and calluses using a chemical agent or plaster. They should not be excised at home and must be managed by trained staff.
- Always wear socks with shoes and check inside shoes for foreign objects before wearing them.
- Avoid walking barefoot at all times.
- Ensure a qualified healthcare provider examines your feet regularly.
- Notify the healthcare provider at once if a blister, cut, scratch, or sore develops.

Evidence for the effectiveness of patient education on foot care is lacking. A Cochrane review of 11 randomised controlled trials concluded that brief foot care education alone does positively influence patient knowledge and behaviour in the short term, but it is ineffective in preventing diabetic foot ulcers. Education in a structured, organised, and repetitive manner, combined with preventive interventions may, however, prevent foot problems.^29^ Although the International Working Group on the Diabetic Foot acknowledges the limited evidence on long term efficacy of patient education, it recommends some form of patient education to improve their foot care knowledge and behaviour.^10^

### Footwear

Occlusive footwear causes sweating and can predispose to fungal infection,^30^ ^31^ particularly in tropical countries. Ideally, footwear for people with diabetes should have a wide toe box, soft cushioned soles, extra depth to accommodate orthoses if required, and laces or Velcro for fitting and adjustments. A new pair of shoes can be worn for a short while daily until comfortable. Patient compliance to prescribed footwear is usually poor, particularly at home where they are more active.^29^ Patients with plantar ulcers at forefoot or heel may be offered offloading footwear (fig 5) to allow ulcer healing and prevent recurrence.

**Figure f5:**
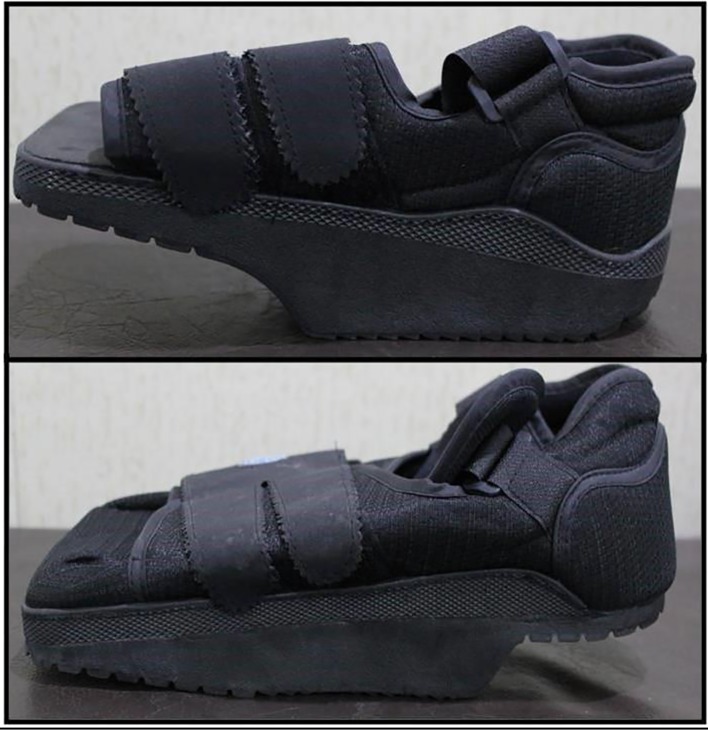
**Fig 5** Offloading footwear reduces pressure on a specific part of the foot to allow an ulcer on that part to heal or to prevent new ulcers. The top figure shows footwear that reduced pressure on the forefoot and the footwear shown underneath allows pressure on the heel to be offloaded

## When to refer?

Refer immediately patients with a life threatening or limb threatening problem such as foot ulceration with fever or any signs of sepsis; ulceration with limb ischaemia; gangrene, or a suspected deep seated soft tissue or bone infection usually indicated by either a grossly swollen foot with shiny skin and patches of discoloration or a gritty feel to the bone during a probe to bone test in an open wound.^9^ Refer to a specialised diabetic foot centre or to general surgery for wound care, offloading, revascularisation if needed, and rehabilitation.

Explain to patients the need to seek specialist care to limit complications. Provide detailed and clear communication before patients are referred so that multidisciplinary care can be facilitated at the earliest opportunity.

Before referral, wash the ulcer with clean water or saline and apply a sterile inert dressing such as a saline soaked gauze to control exudates and maintain a warm, moist environment for healing. Avoid microbicidal agents such as hydrogen peroxide, povidone iodine, or chlorhexidine to clean or dress the ulcer as these are cytotoxic. Costly antimicrobial dressings are not recommended.^9^ Adjust dressings, footwear, and ambulation to avoid weight bearing on an ulcerated foot.^32^ Early and aggressive treatment to control infection is important, especially in the presence of an ulcer. Start antibiotic treatment according to antibiotic policy based on local resistance patterns. Before starting antibiotics, take a piece of soft tissue from the base of the ulcer for culture and sensitivity, or take a deep swab for culture.^9^ Refer urgently, within one or two days, patients with a history of rest pain, uncomplicated ulcer, or acute Charcot foot.^9^ For patients with rest pain or intermittent claudication, offer referral to vascular intervention services for further investigations such as Duplex ultrasonography, and consideration for revascularisation.^21^

The management and referral pathways between primary care, specialty diabetic foot centres, and multidisciplinary foot care services need to be integrated (see infographic).

**How can diabetic foot care services be organised in India?**

Nearly 415 million people globally have diabetes, with 75% living in low and middle income countries. In India about 70 million people have diabetes, and the number is projected to rise to 125 million by 2040.^34^

The National Institute for Health and Care Excellence guideline on diabetic foot recommends a three tier system for foot care: primary healthcare for preventive services and appropriate referral of diabetic foot; foot protection services at community level for podiatric care and management of simple foot problems; and multidisciplinary foot care services at tertiary level to handle complex foot problems.^9^

In low and middle income countries, primary care doctors are not trained in diabetic foot care, podiatry as a discipline is emerging, and multidisciplinary foot care services are available at few tertiary care centres.

We recommend training primary care doctors in diabetic foot care, particularly in countries with a high burden of diabetes. Referral hospitals should develop diabetic foot centres under the specialty of general surgery. These centres would provide foot protection services such as callus debridement and nail care, and surgeries such as wound debridement and minor or major amputations. Multidisciplinary foot care services should be provided at all tertiary level hospitals with facilities for vascular intervention and orthoses.

**Education into practice**

- In your practice, what proportion of people with diabetes have had a foot evaluation in the past 12 months?
- Describe how you would screen patients with diabetes for peripheral neuropathy and peripheral arterial disease.
- How would you advise a patient with diabetes about foot care?

**How patients were involved in the creation of this article**

No patients were involved in the creation of this article.

**Additional resources**

**For healthcare providers**

- Indian Ministry of Health and Family Welfare. Standard treatment guidelines: The diabetic foot: prevention and management in India, 2016. http://clinicalestablishments.nic.in/En/1068-standard-treatment-guidelines.aspx http://clinicalestablishments.nic.in/WriteReadData/5381.pdf
- International Working Group on the Diabetic Foot. Guidance on footwear and offloading interventions to prevent and heal foot ulcers in people with diabetes. www.iwgdf.org/files/2015/website_footwearoffloading.pdf
- National Institute for Health and Care Excellence clinical guideline on diabetic foot problems: prevention and management, 2015. www.nice.org.uk/guidance/ng19/chapter/1-recommendations
- National Institute for Health and Care Excellence clinical guideline on peripheral arterial disease: diagnosis and management 2012, updated 2017. www.nice.org.uk/guidance/cg147.
- Infectious Diseases Society of America clinical practice guideline for the diagnosis and treatment of diabetic foot infections, 2012. https://academic.oup.com/cid/article-lookup/doi/10.1093/cid/cis346

For patients*

- NHS Choices. Diabetes. www.nhs.uk/Conditions/Diabetes/Pages/Diabetes.aspx
- NHS Choices. How to look after your feet if you have diabetes. www.nhs.uk/Livewell/foothealth/Pages/Diabetesandfeet.aspx
- NHS Choices. Why feet sensations are lost and how to take care of them. www.nhs.uk/Conditions/Peripheral-neuropathy/Pages/Complications.aspx
- NHS Choices. What does a podiatrist do and how can a podiatrist help you? www.nhs.uk/livewell/foothealth/pages/foot-problems-podiatrist.aspx
- NHS Choices. How do common foot problems look? www.nhs.uk/Tools/Pages/Foot-problems-a-visual-guide.aspx

*All these web links are freely available on the internet.

**Suggestions for future research**

- Does grading the severity of peripheral arterial disease using the ankle brachial index help guide interventions to prevent foot ulcers in people with diabetes?
- What is the sensitivity of the monofilament test to diagnose peripheral neuropathy, and the interobserver variation among trained providers?
- What model of patient education is effective in preventing diabetic foot complications?

## Web Extra

Extra material supplied by the author

Infographic

## Notes

Provenance and peer review: This article is one of a series commissioned by the *BMJ* from the Global Health and Development Group at Imperial College London (formerly NICE International) as part of the International Decision Support Initiative (www.idsihealth.org). The *BMJ* retained full editorial control over external peer review, editing, and publication. Open access fees are funded by the Bill and Melinda Gates Foundation.

Patient consent: All photographs have been included after taking patient consent.

## References

[1] Zhang P, Lu J, Jing Y, Tang S, Zhu D, Bi Y. Global epidemiology of diabetic foot ulceration: a systematic review and meta-analysis (†). Ann Med 2017;49:106-16. 10.1080/07853890.2016.1231932 pmid:27585063. 27585063

[2] Schaper NC, Apelqvist J, Bakker K. The international consensus and practical guidelines on the management and prevention of the diabetic foot. Curr Diab Rep 2003;3:475-9. 10.1007/s11892-003-0010-4 pmid:14611743. 14611743

[3] Jeffcoate W, Bakker K. World Diabetes Day: footing the bill. Lancet 2005;365:1527 10.1016/S0140-6736(05)66437-9 pmid:15866295. 15866295

[4] Lazzarini PA, Hurn SE, Fernando ME, et al. Prevalence of foot disease and risk factors in general inpatient populations: a systematic review and meta-analysis. BMJ Open 2015;5:e008544 10.1136/bmjopen-2015-008544 pmid:26597864. PMC4663442 26597864

[5] Singh N, Armstrong DG, Lipsky BA. Preventing foot ulcers in patients with diabetes. JAMA 2005;293:217-28. 10.1001/jama.293.2.217 pmid:15644549. 15644549

[6] Bhat S, Mary S, Giri AP, Kulkarni MJ. Advanced glycation end products in diabetic complications. In: *Mechanisms of vascular defects in diabetes mellitus*. Springer International, 2017:423-49.

[7] Frykberg RG, Belczyk R. Epidemiology of the Charcot foot. Clin Podiatr Med Surg 2008;25:17-28, v. 10.1016/j.cpm.2007.10.001 pmid:18165108. 18165108

[8] Rogers LC, Frykberg RG, Sanders LJ. The diabetic Charcot foot: recognition, evaluation and management. In: Armstrong DG, Lavery LA, eds. *Clinical care of the diabetic foot*. 3rd ed. 2016: 99.

[9] International Guidelines Team. National Institute for Health and Care Excellence clinical guideline 19. Diabetic foot problems: prevention and management. Updated 2016. www.nice.org.uk/guidance/ng19.

[10] Bus SA, van Netten JJ, Lavery LA, et al. International Working Group on the Diabetic Foot guidance on the prevention of foot ulcers in at-risk patients with diabetes. Diabetes Metab Res Rev 2016;32:16-24. 10.1002/dmrr.2696 pmid:26334001. 26334001

[11] British Columbia Provincial Nursing Skin and Wound Committee. Procedure: Monofilament testing for loss of protective sensation of diabetic/neuropathic feet for adults and children. 2014:1-3. www.clwk.ca/buddydrive/file/procedure-monofilament-testing/?download=106%253Aprocedure-monofilament-testing-for-lops.

[12] Smieja M, Hunt DL, Edelman D, Etchells E, Cornuz J, Simel DL. International Cooperative Group for Clinical Examination Research. Clinical examination for the detection of protective sensation in the feet of diabetic patients. J Gen Intern Med 1999;14:418-24. 10.1046/j.1525-1497.1999.05208.x pmid:10417599. 10417599 PMC1496604

[13] Mythili A, Kumar KD, Subrahmanyam KA, Venkateswarlu K, Butchi RG. A comparative study of examination scores and quantitative sensory testing in diagnosis of diabetic polyneuropathy. Int J Diabetes Dev Ctries 2010;30:43-8. 10.4103/0973-3930.60007 pmid:20431806. 20431806 PMC2859284

[14] Booth J, Young MJ. Differences in the performance of commercially available 10-g monofilaments. Diabetes Care 2000;23:984-8. 10.2337/diacare.23.7.984 pmid:10895851. 10895851

[15] Mayfield JA, Sugarman JR. The use of the Semmes-Weinstein monofilament and other threshold tests for preventing foot ulceration and amputation in persons with diabetes. J Fam Pract 2000;49(Suppl):S17-29.pmid:11093555. 11093555

[16] Dros J, Wewerinke A, Bindels PJ, van Weert HC. Accuracy of monofilament testing to diagnose peripheral neuropathy: a systematic review. Ann Fam Med 2009;7:555-8. 10.1370/afm.1016 pmid:19901316. 19901316 PMC2775618

[17] Pham H, Armstrong DG, Harvey C, Harkless LB, Giurini JM, Veves A. Screening techniques to identify people at high risk for diabetic foot ulceration: a prospective multicenter trial. Diabetes Care 2000;23:606-11. 10.2337/diacare.23.5.606 pmid:10834417. 10834417

[18] Vijay V, Snehalatha C, Seena R, Ramachandran A. The Rydel Seiffer tuning fork: An inexpensive device for screening diabetic patients with high-risk foot. Pract Diabetes Int 2001;18:155-6 10.1002/pdi.170.

[19] Hinchliffe R, Brownrigg J, Apelqvist J, et al. International Working Group on the Diabetic Foot guidance on the diagnosis, prognosis and management of peripheral artery disease in patients with foot ulcers in diabetes. Diabetes Metab Res Rev 2016;32:37-44. 10.1002/dmrr.2698 pmid:26332424. 26332424

[20] Damir A. Clinical assessment of diabetic foot patient. J Int Med Sci Acad 2011;24:199-203.

[21] National Institute for Health and Care Excellence. Peripheral arterial disease: diagnosis and management. Guideline 147, 2012. www.nice.org.uk/guidance/cg147. 32073808

[22] American Diabetes Association. Epidemiology and impact of peripheral arterial disease in people with diabetes. Diabetes 2003;26:3333-41. 10.2337/diacare.26.12.3333 14633825

[23] Mohler ER 3rd, , Treat-Jacobson D, Reilly MP, et al. Utility and barriers to performance of the ankle-brachial index in primary care practice. Vasc Med 2004;9:253-60. 10.1191/1358863x04vm559oa pmid:15678616. 15678616

[24] Haigh KJ, Bingley J, Golledge J, Walker PJ. Barriers to screening and diagnosis of peripheral artery disease by general practitioners. Vasc Med 2013;18:325-30. 10.1177/1358863X13505673 pmid:24105616. 24105616

[25] Chaudru S, de Müllenheim P-Y, Le Faucheur A, Kaladji A, Jaquinandi V, Mahé G. Training to perform ankle-brachial index: systematic review and perspectives to improve teaching and Learning. Eur J Vasc Endovasc Surg 2016;51:240-7. 10.1016/j.ejvs.2015.09.005 pmid:26602321. 26602321

[26] Ang L, Jaiswal M, Martin C, Pop-Busui R. Glucose control and diabetic neuropathy: lessons from recent large clinical trials. Curr Diab Rep 2014;14:528 10.1007/s11892-014-0528-7 pmid:25139473. 25139473 PMC5084623

[27] Pop-Busui R, Boulton AJM, Feldman EL, et al. Diabetic neuropathy: A position statement by the American diabetes association. Diabetes Care 2017;40:136-54. 10.2337/dc16-2042 pmid:27999003. 27999003 PMC6977405

[28] American Diabetes Association. Standards of medical care in diabetes—2017. Abridged for primary care providers. Clin Diabetes 2017;35:5-26.pmid:28144042. 28144042 10.2337/cd16-0067 PMC5241768

[29] Dorresteijn JAN, Valk GD. Patient education for preventing diabetic foot ulceration. Diabetes Metab Res Rev 2012;28(Suppl 1):101-6. 10.1002/dmrr.2237 pmid:22271733. 22271733

[30] Thomas J, Jacobson GA, Narkowicz CK, Peterson GM, Burnet H, Sharpe C. Toenail onychomycosis: an important global disease burden. J Clin Pharm Ther 2010;35:497-519. 10.1111/j.1365-2710.2009.01107.x pmid:20831675. 20831675

[31] Ameen M. Epidemiology of superficial fungal infections. Clin Dermatol 2010;28:197-201. 10.1016/j.clindermatol.2009.12.005 pmid:20347663. 20347663

[32] Lipsky BA, Berendt AR, Cornia PB, et al. Infectious Diseases Society of America clinical practice guideline for the diagnosis and treatment of diabetic foot infections. Clin Infect Dis 2012;54:132-73 10.1093/cid/cis346. 22619242

[33] Ministry Health and Family Welfare of India. Standard treatment guidelines: The diabetic foot: prevention and management in India, 2016. Ministry Health and Family Welfare, India. 2016. www.nhm.gov.in/nrhm-instate/520-standard-treatment-guidelines.html

[34] International Diabetes Federation. IDF diabetes atlas—2015, 7th ed. IDF, 2015. www.diabetesatlas.org/resources/2015-atlas.html. 35914061

