# Supplementary material for: Diabetic foot
Source: BMJ. 2017 Nov 17;359:j5064. doi: 10.1136/bmj.j5064 (PMC5688746; doi:10.1136/bmj.j5064)

thebmj Visual summary

Diabetic foot

Primary care assessment and monitoring

General assessment

Look for signs of sepsis: Visibly unwell · Drowsy · Abnormal breathing · Abnormal pulse · Fever → Urgent referral

Foot examination

Check for active disease: Ulceration · Rest pain · Gangrene · Cellulitis → Urgent referral

Check foot temperature and colour
- Cold, pale or dusky → May indicate ischaemia (ISC) → Acute limb or life threatening problems
- Warm, red or swollen → May indicate acute Charcot foot (CHA) → Acute limb or life threatening problems

Acute limb or life threatening problems → Urgent referral

DEF — Check for lesions and deformities: Deformed nails · Callus · Macerated web spaces · Skin fissures · Hallux valgus · Claw toes · Hammer toes · Pes cavus · Rocker bottom foot · Fungal infection

Urgent referral to diabetic foot centre or general surgery

PAD — Screen for peripheral arterial disease

- Absent foot pulses: Posterior tibial artery · Dorsalis pedis artery
- History of intermittent claudication
- Ankle brachial index (ABI) less than 0.9 — Measure if possible

LOPS — Screen for loss of protective sensation

Test sensation with a 10 g monofilament: An inability to sense a 10 gram pressure is the current consensus definition of LOPS

± Biothesiometer

± Graduated tuning fork

Risk assessment

LOW RISK
CAL — Callus alone

MEDIUM RISK
Deformity, loss of protective sensation, or peripheral arterial disease
DEF · LOPS · PAD

HIGH RISK
Previous amputation or ulceration (PRE)
Any two of: loss of protective sensation, peripheral arterial disease, and lesions or deformities (LOPS + PAD + DEF)

Patient education
- Glycaemic control
- When to seek help
- Modifiable risk factors
- Foot care

Manage PAD*
- Statins + 1 antiplatelet
- Exercise to improve circulation
- Consider referral for further investigations and revascularization

Routine referral · Urgent referral

DIABETIC FOOT CENTRE
- Foot protection services: Callus debridement · Nail care
- Surgical management: Wound debridement · Amputations

Liaison or referral → Vascular intervention and orthoses services

* Adapted from NICE guidance on diabetic foot and peripheral arterial disease

Primary care follow-up

LOW RISK	MEDIUM RISK	HIGH RISK
Every year	Every 3–6 months	Every 1–2 months

thebmj Read the full article online http://bit.ly/BMJdfoot

© 2017 BMJ Publishing group Ltd.

Disclaimer: This infographic is not a validated clinical decision aid. This information is provided without any representations, conditions, or warranties that it is accurate or up to date. BMJ and its licensors assume no responsibility for any aspect of treatment administered with the aid of this information. Any reliance placed on this information is strictly at the user's own risk. For the full disclaimer wording see BMJ's terms and conditions: http://www.bmj.com/company/legal-information/

See more visual summaries http://www.bmj.com/infographics

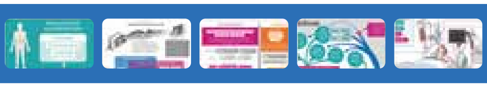

Supplement: Supplementary file 1 — Infographic [file chas041916.ww1.pdf]
